# Supplementary material for: Stacking Model‐Based Classifiers for Dealing With Multiple Sets of Noisy Labels
Source: Biom J. 2025 Mar 12;67(2):e70042. doi: 10.1002/bimj.70042 (PMC11898607; doi:10.1002/bimj.70042)
Supplement: Supplementary file 1 — Supporting Information [file BIMJ-67-e70042-s001.zip › density-based-ensemble-model-main/competitors_real_data/netlab-master/netlab-master/help/mdnpost.htm]

Netlab Reference Manual mdnpost


# mdnpost

## Purpose

Computes the posterior probability for each MDN mixture component.

## Synopsis

```
post = mdnpost(mixparams, t)
[post, a] = mdnpost(mixparams, t)
```

## Description

`post = mdnpost(mixparams, t)` computes the posterior
probability `p(j|t)` of each
data vector in `t` under the Gaussian mixture model represented by the
corresponding entries in `mixparams`. Each row of `t` represents a
single vector.

`[post, a] = mdnpost(mixparams, t)` also computes the activations
`a` (i.e. the probability `p(t|j)` of the data conditioned on
each component density) for a Gaussian mixture model.

## See Also

`mdngrad`, `mdnprob`

---

**Pages:**
Index


---

Copyright (c) Ian T Nabney (1996-9)

David J Evans (1998)
